# Shaping Rare Granulomatous Diseases in the Lab: How New Models Are Changing the Game

**DOI:** 10.3390/cells14040293

**Published:** 2025-02-16

**Authors:** Jessica Ceccato, Giulia Gualtiero, Maria Piazza, Samuela Carraro, Helena Buso, Carla Felice, Marcello Rattazzi, Riccardo Scarpa, Fabrizio Vianello, Francesco Cinetto

**Affiliations:** 1 Hematology and Clinical Immunology Unit, Department of Medicine (DIMED), University of Padua, 35128 Padua, Italy; jessica.ceccato.1@phd.unipd.it (J.C.); giulia.gualtiero@studenti.unipd.it (G.G.); 2 Veneto Institute of Molecular Medicine (VIMM), 35128 Padua, Italy; 3 Department of Medicine (DIMED), University of Padua, 35128 Padua, Italy; maria.piazza@unipd.it (M.P.); helena.buso@aulss2.veneto.it (H.B.); carla.felice@unipd.it (C.F.); marcello.rattazzi@unipd.it (M.R.); riccardo.scarpa@unipd.it (R.S.); francesco.cinetto@unipd.it (F.C.); 4 Rare Diseases Referral Center, Internal Medicine 1, Ca’ Foncello Hospital, AULSS2 Marca Trevigiana, 31100 Treviso, Italy; samuela.carraro@unipd.it

**Keywords:** granuloma, sarcoidosis, interstitial lung disease, 3D models, multinucleated giant cells

## Abstract

In vitro models serve as valuable tools for understanding the complex cellular and molecular interactions involved in granuloma formation, providing a controlled environment to explore the underlying mechanisms of their development and function. Various models have been developed to replicate granulomatous diseases, even though they may lack the sophistication needed to fully capture the variability present in clinical spectra and environmental influences. Traditional cultures of PBMCs have been widely used to generate granuloma models, enabling the study of aggregation responses to various stimuli. However, growing cells on a two-dimensional (2D) plastic surface as a monolayer can lead to altered cellular responses and the modulation of signaling pathways, which may not accurately represent in vivo conditions. In response to these limitations, the past decade has seen significant advancements in the development of three-dimensional (3D) in vitro models, which more effectively mimic in vivo conditions and provide better insights into cell–cell and cell–microenvironment interactions. Meanwhile, the use of in vivo animal models in biomedical research must adhere to the principle of the three Rs (replacement, reduction, and refinement) while ensuring that the models faithfully replicate human-specific processes. This review summarizes and compares the main models developed to investigate granulomas, focusing on their contribution to advancing our understanding of granuloma biology. We also discuss the strengths and limitations of each model, offering insights into their biological relevance and practical applications.

## 1. Introduction

A granuloma represents an organized structure composed of mature macrophages, multinucleated giant cells (MGCs), activated monocytes, T and B lymphocytes, and fibroblasts, which can potentially develop in any district of the body [1,2]. It is well-established that granuloma formation is associated with the activation of both innate and adaptive immunity [3]. Specifically, an antigen can trigger a strong immune response, leading to the recruitment of macrophages and T-helper (Th) lymphocytes [4]. These cells surround the antigen to create a physical barrier, protecting adjacent tissues from damage. The inability to eradicate the antigen or deactivate the triggering factors is a key characteristic of granuloma formation [3].

Granuloma formation can be induced by various microorganisms, including Schistosomes [5], Cryptococcus [6], Dermatophytes [7], and Mycobacteria [8] (e.g., *Mycobacterium tuberculosis*), as well as by exposure to silica crystals, tattoo ink, wood powder, and carbon nanotubes [9,10]. However, granulomas can also form without a known trigger, as in sarcoidosis [8,11] or common variable immunodeficiency (CVID) [12].

Sarcoidosis is a rare multisystem inflammatory disease that can affect individuals of any age, ethnicity, or sex, with a slight predominance in women. Its onset may be influenced by environmental exposure and socioeconomic factors, contributing to significant variability in its global prevalence and clinical presentation [13]. Sarcoidosis is characterized by the development of non-caseating granulomas, typically in the lungs, but it can also affect extrapulmonary sites. Symptoms and the impact on patients’ quality of life depend on the organs involved [14,15]. While sarcoidosis has been associated with exposure to both infectious and non-infectious agents, the precise mechanisms underlying its development and persistence remain unclear. After granulomas form, some sarcoidosis patients experience spontaneous remission, whereas approximately 30–50% develop chronic or treatment-resistant disease. The factors determining the transition between spontaneous resolution and chronic progression are not yet understood [1].

Non-necrotizing granulomas are a hallmark of sarcoidosis, though they are neither specific nor pathognomonic. Interestingly, recent studies have shown that granulomas can form in patients with defects in adaptive immunity, highlighting the importance of innate immunity in granuloma development [3,16,17]. Notably, about 20% of CVID patients develop granulomatous lymphocytic interstitial lung disease (GLILD), a non-infectious interstitial lung disease characterized by non-necrotizing granulomas and associated with a worse prognosis; granulomatous disease may also occur as a non-infectious complication of other inborn errors of immunity (IEI) [18].

In contrast with sarcoidosis and GLILD, tuberculosis is marked by necrotizing granulomas, with a well-established etiology [19,20]. Tuberculous granulomas are directly induced by *Mycobacterium tuberculosis* infection, and their formation mechanisms have been extensively studied [19,20]. Despite some overlapping clinical, molecular, and immunological features that might suggest tuberculosis and sarcoidosis represent two ends of the same spectrum [21], their clinical and biological differences are clear [22]. GLILD presents an even more distinct case, where granulomas partially resembling those of sarcoidosis arise in the context of underlying immunodeficiency [23].

**Figure 1:**
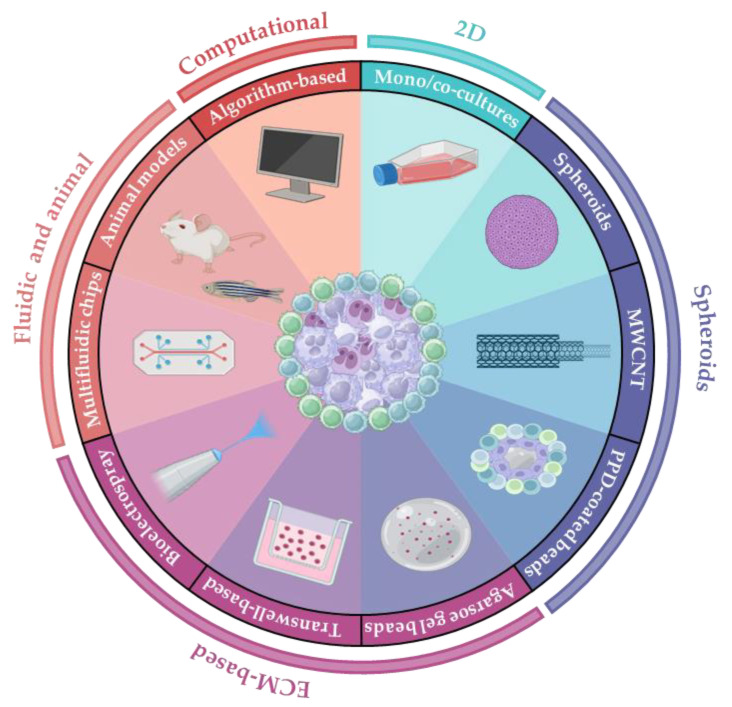
Graphical representation of available granuloma experimental models. From the top, in clockwise order: bi-dimensional mono- and co-culture systems; spheroids induced by *M. bovis* BCG infection; tridimensional models induced by MWCNTs; PPD-coated bead-induced models; extracellular-based models involving the use of agarose gel beads; transwell-based models; bioelectrospray-based models; fluidic systems and biochips; animal models; computational models. See Table 1 for details. Partially created in BioRender.

Because the mechanisms of tuberculosis granuloma formation are better understood [24], many existing in vitro and in vivo granuloma models are based on *M. tuberculosis* infection. Over the years, however, other models of granuloma formation have been proposed, with the aim of recapitulating this type of inflammation unrelated to the mycobacterial infection trigger. The aim of this review is to summarize the existing granuloma models (Table 1; Figure 1) and discuss the strengths and limitations of these models in replicating different types of granulomas, particularly those occurring in diseases where the causative agents remain unidentified, such as sarcoidosis and GLILD.

## 2. Available Models for Granulomas Studies

### 2.1. Two-Dimensional Models: MGC Induction Strategies

Despite their simplified nature, MGC induction models naturally serve as the initial starting point in the development of a granuloma model. Numerous protocols have been developed for inducing in vitro MGC formation using diverse combinations of natural mitogens, cytokines, pathogens, parasites, and chemical agents employed for MGC induction (Table 2).

#### 2.1.1. Natural Mitogens

One of the earliest and simplest in vitro models for investigating sarcoidosis granuloma formation was proposed in 2001 by Mizuno and colleagues, based on the presence of MGCs, a key hallmark of granuloma structure. In their study, monocytes isolated from sarcoidosis patients and healthy donors were cultured with a conditioned medium containing the supernatant of concanavalin A-stimulated peripheral blood mononuclear cells (PBMCs). Notably, cells derived from sarcoidosis patients demonstrated a greater ability to form MGCs compared with those from healthy controls [35].

A similar approach was later adopted by Yanagishita et al., who treated the RAW mouse macrophage cell line with lipopolysaccharide (LPS) and concanavalin A. This co-treatment induced the formation of MGCs and was associated with a marked increase in the levels of TNF-α, a key mediator and therapeutic target in sarcoidosis granuloma [36].

#### 2.1.2. Cytokines Combinations

In 1984, Weineberg and colleagues demonstrated that MGCs could be induced in vitro by incubating monocytes with IFN-γ. These MGCs exhibited increased hydrogen peroxide (H_2_O_2_) production when exposed to phorbol myristate acetate (PMA) [37]. A few years later, in 1990, MGCs were successfully generated from cells cultured with supernatants derived from concanavalin A-stimulated PBMCs or through direct treatment with IFN-γ. Interestingly, other monocyte-activating cytokines, such as IL-2, neither independently induced MGC formation nor enhanced IFN-γ activity. Of note, the frequency of MGC formation was higher with the supernatant treatment alone compared with the IFN-γ treatment [38,39].

In 1992, Enelow and colleagues tested several cytokine combinations and found that the pairing of IL-3 and IFN-γ was the most effective, achieving 67 ± 6% cell fusion within one week without additional stimuli. Fusion was defined as the ratio of nuclei within MGCs to the total number of nuclei in the culture [40]. This study highlighted that monocytes undergo two sequential steps for fusion and differentiation, first involving adherence-promoting factors, followed by fusion-promoting agents.

A significant advancement came from McNally and Anderson [41], who developed a two-step protocol for inducing macrophage morphological development and MGC formation over ten days. Their method involved the initial stimulation of primary monocytes adhered to plasticware with granulocyte-macrophage colony-stimulating factor (GM-CSF) to maximize cell density, followed by stimulation with IL-4 to induce macrophage fusion. This combination proved to be more effective than untreated monocytes. They also demonstrated that while IL-3 could induce fusion similarly to GM-CSF, it failed to produce large giant cells. This work was pivotal in identifying IL-4 as a potent macrophage fusion factor when paired with GM-CSF.

Building on this approach, Dugast and colleagues refined the protocol in 1997 by introducing a seven-day GM-CSF treatment to promote monocyte differentiation, followed by IL-4 stimulation to drive MGC formation [25]. Furthermore, macrophage colony-stimulating factor (M-CSF) alone or in combination with IL-13 or IL-4 was also reported to induce time-dependent MGC formation in vitro. However, this approach predominantly produced osteoclast-like cells, which are not representative of granuloma-associated MGCs [42,43]. Additionally, GM-CSF and IL-4 treatment has been employed to generate MGCs from monocytes derived from CVID patients and currently represents the only available attempt to study GLILD in vitro modeling [48].

More recently, Lim and colleagues proposed an optimized strategy for inducing sarcoid-sorted macrophage differentiation into MGCs and clusters without using beads or microbial agents/antigens. In particular, sarcoid monocyte-derived macrophages from chronic sarcoidosis patients were autonomously able to generate large aggregates under GM-CSF treatment, similar to the ones observed in primitive granulomas. Their method emphasized the critical role of GM-CSF in the early stages of in vitro stimulation as a macrophage differentiation factor [49]. Moreover, RNA-seq performed on monocytes-derived macrophages from chronic sarcoidosis patients revealed an enrichment of lipid metabolism-related pathways, such as the ones involved in cholesterol homeostasis and the SREBP control of lipid synthesis. This upregulation was combined with the hyperactivation of the mTORC1 signaling pathway and the IFN-γ response [49].

Interestingly, in vitro MGC differentiation is predominantly driven by cytokines associated with a Th2 microenvironment, particularly IL-4 and IL-13. This highlights the importance of cell–cell interactions in granuloma induction and maintenance. Moreover, Th2 cytokines are implicated in the polarization of M2 macrophages, further highlighting their relevance in granuloma biology and the promotion of fibrosis [50].

#### 2.1.3. Bacteria and Parasites

MGCs have also been successfully generated in vitro by using pathogens or parasites, with *Mycobacterium tuberculosis* being the most commonly employed agent. In 1999, Gasser and Möst demonstrated MGC formation by stimulating monocytes with *Mycobacterium bovis* bacillus Calmette–Guérin (BCG) in the presence of cytokine-containing supernatants derived from *Herpesvirus saimiri*-transformed human T-cell clones. This approach resulted in a fusion rate of 27% [44].

Another notable example comes from Seitzer and colleagues, who induced MGC formation by co-culturing human PBMCs with *Nippostrongylus brasiliensis* larvae, a gastrointestinal roundworm. Over 7 to 14 days, the monocytes differentiated into mature macrophages, which subsequently formed epithelioid cells and MGCs. These cells clustered around the nematode in a granuloma-like architecture [45].

*Propionibacterium acnes* has been implicated in the development of sarcoidosis as it has been isolated from the lymph nodes of patients with sarcoidosis and localized within sarcoidosis granulomas [51]. To our knowledge, the first in vitro model induced by *P. acnes* was developed in 2017 by Aubin G. G. and colleagues [52]. The model interestingly provides new evidence about the role of that pathogen in granuloma occurrence by showing granuloma-like structure formation after *P. acnes* infection of PBMCs, also leading to a CD4+ or CD8+ T-cell immune response according to the bacterial strain.

Interestingly, much like cytokine-driven induction, the formation of MGCs through pathogens and parasites often leverages Th2-mediated inflammation. Th2 cells play a central role in immune responses triggered by parasites, emphasizing the importance of this pattern of inflammation in granuloma formation and persistence.

#### 2.1.4. Chemical Treatments

Pivotal studies have demonstrated that freshly isolated human monocytes can be induced to form MGCs through treatment with phytohemagglutinin (PHA) or sodium metaperiodate (NaIO_4_). However, the resulting MGCs’ morphology differed from that of Langhans cells typically observed at sites of delayed hypersensitivity reactions [46].

In 1989, Hassan and colleagues developed a protocol utilizing PMA, a protein kinase C activator known to promote cellular proliferation and survival. Adding PMA to three-week-old primary monocyte cultures successfully induced MGC formation, with fusion rates increasing from 30% to 80%. This effect was further enhanced by pretreating monocytes with IFN-γ [47].

Although these two-dimensional (2D) models are relatively simple to establish and manage, they inherently oversimplify the complexity of granuloma architecture and may not fully represent the structural and functional characteristics of the physiological context observed in vivo. Nevertheless, they offer a solid basis for studying granuloma-related processes and could potentially be applied across various granulomatous diseases.

### 2.2. Three-Dimensional In Vitro Models

The development of animal-free models in biomedical research is strongly encouraged by the 3Rs conception: “*replacement, reduction, refinement*”. In particular, replacement refers to technologies or approaches that may replace the use of animals in experiments. The development of three-dimensional models has proved useful in various experimental situations. Here, we will discuss 3D models that have proved to be effective in defining the pathogenesis of granulomas.

#### 2.2.1. Spheroids Models

Among three-dimensional (3D) tuberculosis granuloma models, one of the most noteworthy was reported in 2021 by Mukundan and colleagues. They developed a matrix-free 3D model using THP-1 human monocyte/macrophage cells [26]. THP-1 cells were cultured with *M. bovis* BCG in ultra-low-attachment plates, allowing the cells to self-aggregate and form 3D inflammatory spheroids that gradually increased in size. Notably, bacterial presence was observed exclusively within the macrophages of the spheroids, with no bacilli detected outside the cells.

The study highlighted significant differences between spheroids generated from activated macrophages and those formed by non-activated macrophages. This model provides a valuable tool for studying mycobacterial-induced granulomas and screening anti-mycobacterial treatments. However, its relevance to granulomas induced by non-microbial factors may be limited as it is not fully applicable to the study of sarcoidosis and GLILD. In fact, as previously reported, sarcoidosis and GLILD involve different underlying immune mechanisms (e.g., dysregulated immune responses, T-cell activation, epithelial cell interactions, and environmental factors), which are not directly captured by a mycobacterial infection model and unable to properly recreate the non-caseating granulomatous microenvironment [46]. Moreover, recent histological evaluations combined with spatial proteomics and immunofluorescent analyses performed on tissue sections coming from GLILD, sarcoidosis, tuberculosis, and pseudotuberculosis patients have underlined a distinct pathophysiological setting, as peculiar cellular and histological organization has been observed in CVID granulomas, marked by an increased influx of both myeloid and lymphoid cells. Indeed, granulomas in GLILD patients appear to be less well-circumscribed, less fibrotic, and more organized in clusters, in contrast with what is observed in sarcoidosis, tuberculosis, and pseudotuberculosis samples [23]. Hence, it is now clear how mycobacterial-induced 3D spheroid models show greater biological limitations, which underscores the need for more comprehensive in vitro platforms able to recapitulate the complex and distinct pathophysiological microenvironments of non-tuberculous granulomatous diseases.

Interestingly, granulomas can also be triggered by inorganic agents, such as multi-walled carbon nanotubes (MWCNTs). When monocytes or macrophages are exposed to nanotubes, they form stable aggregates exhibiting granuloma-like characteristics, including the presence of MGCs and the expression of pro-inflammatory and pro-fibrotic markers [53]. In this regard, Kala and colleagues successfully set up an in vitro human granuloma model as an advanced platform for investigating pathogenesis, observing patient-specific responses, and performing early drug screening for treating granulomatous disease, most specifically sarcoidosis, as environmental exposure is considered among the risk factors. In particular, PBMCs collected from healthy donors were subjected to a sublethal dose of MWCNTs for 7 days. MWCNT stimulation led to the formation of granuloma-like structures resembling traits observed in bronchoalveolar lavage cytologic samples and tissue granulomas coming from sarcoidosis patients and characterized by a peculiar cytokine secretion profile. Indeed, the PBMCs’ transcriptomic profile revealed enrichment in the gene set involving the JAK-STAT and TNF signaling pathways, widely acknowledged as fundamental in granuloma development and maintenance [27].

This knowledge has also been applied to develop in vitro 3D spheroids using MWCNTs, as shown by Barna and colleagues [9]. Interestingly, the formation of epithelioid granulomas appears to be more strongly linked to the shape and 3D structure of MWCNTs than to their iron content or surface area. This might suggest that the geometric properties of these triggers, rather than their chemical composition and overall size, may play a more significant role in triggering granuloma formation. These findings throw light on the importance of considering MWCNTs’ morphology when evaluating their biological effects, particularly in terms of immune system activation and granulomatous inflammation.

Models based on MWCNTs offer time- and cost-effective platforms for studying granuloma formation associated with nanomaterial inhalation, including carbon nanotubes, nanofibers, nanorods, and metallic nanowires, in the absence of microbial agents and exogenous cytokines. However, due to the specificity of the trigger, it remains unclear whether these models represent only exposure-related granulomas or if they can be generalized to other types, such as sarcoidosis and GLILD granulomas.

In vitro 3D spheroid induction has also been explored using coated beads to promote cell aggregation. In 2017, Crouser and colleagues cultured PBMCs from sarcoidosis patients with uncoated polystyrene beads or beads coated with tuberculin-purified protein derivative (PPD) [28]. Robust multicellular aggregation was observed with the PPD-coated beads in sarcoidosis PBMCs compared with healthy controls, forming a distinct macrophage core surrounded by a lymphocyte shell, as revealed by immunofluorescence assays. In contrast, uncoated beads induced negligible aggregation.

The study marked a significant advancement in modeling the early phases of granuloma formation, soon becoming one of the gold standards in the field; however, the extent of its clinical relevance is still under investigation. The PPD-coated bead model was further used by Locke and colleagues, who correlated aggregation with the differential expression of 1274 genes compared with healthy donors. Interestingly, they observed an IL-13-dependent overexpression of *STAT6*, reflecting a Th2-mediated inflammatory response that drives M2 macrophage polarization, as found in tissue samples of sarcoidosis granuloma. In the same model, increased mTORC1/S6/STAT3 signal transduction was also demonstrated in granulomas obtained from PBMCs of active pulmonary sarcoidosis patients [54].

Another significant contribution came from the subsequent work by the same research group, who pre-clinically tested the use of a potential new drug for sarcoidosis treatment, XTMAB-16, by using PBMCs from active pulmonary sarcoidosis patients and the PPD-coated beads model. XTMAB-16 was shown to inhibit granuloma formation and suppress IL-1β secretion, thus supporting the results of a phase 1 clinical trial (NCT04971395) and opening the way for a currently ongoing phase 1b/2 proof-of-concept study in patients with pulmonary sarcoidosis with or without extrapulmonary manifestations (NCT05890729) [55].

While these models provide valuable insights into granuloma initiation and closely resemble what is happening in pulmonary sarcoidosis, they still rely on mycobacterial derivatives. As such, they may not fully represent the diversity of granuloma types; accordingly, in the same study by Crouser et al., PBMCs from only half of the selected sarcoidosis patients were shown to be high responders to PPD and able to produce significant granuloma-like cell aggregates under this specific exposure [54]. This constraint is in line with the extreme variability of exposure history reported in sarcoidosis patients and may prevent them from fully representing all granuloma types and all features of granulomatous inflammation occurring in sarcoidosis.

#### 2.2.2. Extracellular Matrix Models

One of the earliest 3D models incorporating extracellular matrix components was developed in 1995 by Franklin et al. [30]. They cultured PBMCs in a 3D milieu of agarose beads and collagen gel, facilitating the formation of monocyte/macrophage aggregates that mimicked the early stages of granuloma formation [29]. Significant advancements since then have led to the development of more sophisticated 3D systems.

In 2015, Braian and colleagues developed an innovative transwell-based model featuring a collagen matrix layer overlaid with monocytes/macrophages and pulmonary epithelial cells [30]. Air exposure stimulated the epithelial cells to produce an extracellular matrix (ECM), effectively integrating all cell types into a cohesive structure. When *M. tuberculosis*-infected primary macrophages were introduced, the model demonstrated immune cell migration into the infected tissue, leading to the formation of granuloma-like structures. While groundbreaking, this model is specifically designed for *M. tuberculosis* infection and the lung environment, which limits its applicability to other types of granulomas.

Recognizing the critical role of spatial configuration in cellular growth, Workman et al. proposed a 3D model utilizing microspheres created through bioelectrospray technology to generate hydrogel droplets. These droplets encapsulated monocytes/macrophages along with ECM components [31]. Cells cultured within this enclosed environment exhibited altered behavior compared with traditional cultures, including enhanced protein secretion and changes in gene expression. This approach generated a microenvironment closely resembling physiological conditions. While bioelectrospray offers significant potential as a tool for granuloma modeling, its adoption is hindered by high costs, the need for specialized equipment, and the requirement for highly trained personnel.

#### 2.2.3. Fluidic Systems and Biochips

Although static systems, primarily based on multiwell plates, remain the most commonly used models in research, it is known that fluidic systems may better replicate in vivo conditions. As a result, translational research is shifting toward the development of dynamic models that enable the continuous circulation of cells and media [56].

In 2021, Calcagno and colleagues introduced an innovative biochip designed to replicate pulmonary granuloma conditions [32]. This model combined a multi-channel fluidic platform, representing a “lung-on-a-chip” device, with a previously developed lung-on-membrane model, consisting of a dual chamber containing normal bronchial epithelial cells and human microvascular endothelial cells [32,57]. Granuloma-like structures were generated by introducing human PBMCs, isolated from peripheral blood samples coming from patients with a confirmed diagnosis of sarcoidosis, encapsulated in granuloma-inducing *Mycobacterium abscessus*-derived microparticles into the air–lung interface of the chip.

This biochip represents a groundbreaking advancement as the first pulmonary granuloma model compatible with artificial intelligence (AI) for automated control. Due to the use of multiple cell types and fluidics in a tissue-specific context, it accurately captures the complexity of in vivo conditions with high reproducibility. The model demonstrated significantly elevated concentrations of IL-1β, IL-6, GM-CSF, and IFN-γ in the granuloma-containing lung chip compared with controls. AI integration enables the autonomous regulation of the testing volume and the precise addition of chemicals, making the model particularly well-suited for mimicking pulmonary sarcoidosis.

Later on, this in vitro model has proven to be helpful as a screening platform for identifying new anti-inflammatory treatments, particularly through the investigation of α -melanocyte-stimulating hormone (α-MSH). As an endogenous neuropeptide expressed in the pituitary gland and part of the melanocortin family, α-MSH is known to exert anti-inflammatory and anti-pyretic effects as it regulates immune responses and controls inflammation at the level of the brain and peripheral organs [58]. Using the lung biochip integrated with the granuloma model developed by Calcagno and colleagues, researchers have demonstrated that α-MSH significantly reduces key inflammatory cytokines, including IL-1β, GM-CSF, type I IFN cytokines (ISG15 and IFN-α), and IL-2 compared with untreated controls. These findings highlight the potential of this model in not just dissecting the cellular microenvironment in lung granulomas but also accelerating the development of targeted anti-inflammatory therapies [59].

While the innovative features of this biochip hold great promise for future drug testing, its design is limited to studying the pulmonary environment. Consequently, it does not address the need for effective models to investigate extra-pulmonary complications.

### 2.3. Animal Models

Since animals, apart from horses [60], do not naturally develop sarcoidosis, universally accepted animal models have been lacking for decades. In the context of immunodeficiency, CD19−/− mice [61] and miR-142 and miR-155 knockout (KO) mice [62] have been used as models for CVID but, to the best of our knowledge, no mouse model effectively mimics GLILD manifestation. Some animal models support the pathogenetic role of microorganisms in granuloma formation. Sarcoid-like pulmonary granulomatosis has been generated in mice subcutaneously immunized with *P. acnes* by using complete Freund’s adjuvant. Interestingly, these granulomas appear to be caused by indigenous *P. acnes* pre-existing in the lower respiratory tract of mice by an influx of *P. acnes*-sensitized CD4+ T cells from the circulation, and antimicrobial administration was effective in suppressing the experimentally induced pulmonary granulomatosis [63,64]. Other authors were able to generate pulmonary granulomas in mouse lungs by intratracheal administration of *P. acnes*, supporting the concept of a bacterial component of sarcoidosis disease pathology [65]. Moreover, *P. acnes*-induced granuloma formation in mice has been shown to be an infective response to the pathogen as it is related to an increase in Th17 cell proportion in the peripheral blood. Interestingly, KO mice with the IL-17A gene failed in granuloma formation [66].

Another interesting model of in vivo pathogen–host interactions generating granulomas has been studied by exploiting the zebrafish model. As embryos and larvae of zebrafish are optically transparent, cell interactions can be visualized in real time. In this model, infection with *Mycobacterium marinum*, a close relative of the human pathogen *Mycobacterium tuberculosis,* led to the formation of macrophage aggregates with pathological characteristics of granulomas. Of relevance, as zebrafish embryos do not have lymphocytes, this result supports the idea that the initiation of granuloma-like aggregates and differentiation of macrophages into epithelioid cells is independent of T lymphocytes [34]. Zebrafish models have also been effective in the identification of energy metabolism regulation, which is required for macrophage protection after *M. tuberculosis* infection [67].

Alternative in vivo models of granuloma have been developed, supporting the pathogenetic role of environmental antigens like MWCNTs. When exposed to oropharyngeal instillation of MWCNTs, mice have been shown to develop granulomas that, at least partially, replicate the histopathological traits and gene expression profiles of sarcoidosis. This observation aligns with findings from in vitro models using the same trigger [68,69,70]. To further investigate the pathological mechanisms underlying disease progression and the development of fibrosis—observed in 20% of sarcoidosis patients and leading to higher morbidity and mortality—Soliman and colleagues developed a murine model of chronic granulomatous inflammation. They achieved this by instilling MWCNTs into myeloid-specific ABCG1 (ATP-binding cassette cholesterol transporter) knockout (KO) mice. The instillation enhanced the transition from granulomatous inflammation to pulmonary fibrosis in ABCG1 KO animals compared with wild-type controls. Although the exact mechanisms require further investigation, increased expression of TGF-β-related signaling molecules, IL-13 and Smad-3, was detected in the lungs of the ABCG1 KO mice, correlating with the transition from inflammation to fibrosis [71]. However, it remains unclear whether the use of MWCNTs as a trigger induces a more trigger-specific granulomatous response, potentially limiting its generalizability to other granuloma types.

The most promising animal model for studying non-infectious granulomas was proposed by Linke and colleagues in 2017 [33]. Conditional myeloid deletion of the *Tsc2* gene, which encodes the tuberin protein (an inhibitor of the mTORC1 complex), resulted in mTORC1 activation in macrophages and the spontaneous formation of granulomas in mice, primarily in the lungs, liver, skin, and lymph nodes. Interestingly, pharmacological mTORC1 inhibition in this model induced apoptosis and completely resolved the granulomas, demonstrating the involvement of the mTORC1 pathway in granulomatous manifestations. This powerful work confirmed the possibility of granuloma formation starting from macrophage activation, and in the absence of an interaction between T cells and a specific antigen, an enhanced alternative (M2-like) polarization of macrophages was also confirmed. Interestingly, the manuscript also highlighted the similarities between mouse results and human biopsies and laid the foundations for a clinical trial that confirmed the efficacy of mTORC1 inhibition in sarcoidosis [33]. Moreover, the involvement of the mTORC1 pathway in extrapulmonary compartments has further been proved by Carlos Bueno-Beti and colleagues through the use of the same *Tsc2* KO mouse for modeling cardiac sarcoidosis [72].

While these findings are significant—particularly given the role of mTOR and autophagy in the pathogenesis of sarcoidosis— available genome-wide association studies (GWAS) have not identified *TSC2* mutations as a risk factor for sarcoidosis [73].

Of note, the above-mentioned T cell-free in vitro granuloma model developed by Lim and colleagues, instrumental in demonstrating that altered lipid metabolism in sarcoidosis macrophages is associated with their predisposition to granuloma formation, was further validated in both human sarcoid skin biopsies and the *Tsc2* KO mouse model [49]. First, sarcoidosis granuloma macrophages with a high neutral lipid content were shown to be present in higher percentages inside skin granulomas of sarcoidosis patients and to highly express SREBF1a/c in comparison with controls. Interferences in the lipid metabolism pathway and its relationship with granulomatous inflammation were subsequently evaluated in the *Tsc2* KO model under a cholesterol-free dietary regimen treated with atorvastatin. Interestingly, this treatment plus the dietary regimen reduced the disease severity in mice with a consequent increased survival. Thus, the development of a pivotal in vitro 2D granuloma model, then validated in both patient skin samples and in an in vivo model, has, from a pathological point of view, further proven how macrophages can autonomously generate granulomas and how cellular metabolism may play a key role while paving the way to new possible clinical applications for sarcoidosis patients [49].

### 2.4. Computational Models

The rapid advancement of technology is opening new perspectives in biomedical research, particularly through the integration of bioinformatic tools, which are instrumental in aiding clinicians and researchers to dissect complex pathologies. In 2013, an early in-silico model based on a Petri net framework was developed to simulate the formation of hepatic granulomas in *Leishmania donovani* infection [74]. Validated against experimental data from mice, this model effectively reproduced the disease progression and highlighted the dominant role of IL-10 in the granuloma core. However, its utility is limited to *L. donovani* infections, and its focus on differently activated macrophages (M1 and M2 subtypes) imposes constraints on its broader applicability.

Building on earlier approaches, Hao and colleagues, in 2014, refined a mathematical model to study the dynamics of sarcoidosis in the lungs [75]. This spatial model accounted for granulomas as evolving regions over time and incorporated variables such as the density of macrophages, Treg, Th1, and Th17 cells, as well as concentrations of IL-2, IL-10, IL-12, IL-13, IFN-γ, TNF-α, TGF-β, GM-CSF, and CCL20, along with fluid velocity. Validated using clinical data from lung tissue samples, the model was applied to predict treatment responses to infliximab, anti-IL-12, anti-IFN-γ, and anti-TGF-β. It demonstrated their potential to reduce granuloma growth, providing valuable insights into therapeutic strategies.

More recently, in 2020, Zaharie and colleagues developed a groundbreaking human 3D computational model to investigate the spatial relationships between granulomas, meninges, brain parenchyma, and blood vessels in one of the most severe forms of tuberculosis: tuberculous meningitis (TBM) [76]. Using digital images from 800 histologically stained sections, they reconstructed the 3D structure of granulomas and their connections. This model successfully captured the three types of central nervous system (CNS) granulomas—non-necrotizing, necrotizing granulomatous, and necrotizing abscesses—which represent progressive stages of the same pathological process. The model offers a powerful tool for analyzing granuloma interactions with surrounding CNS tissues and has potential applications in studying tuberculosis and sarcoidosis in other organs. However, its main limitation lies in its dependence on post-mortem samples, as it requires entire organs and adjacent tissues. Consequently, this approach is not yet suitable for designing personalized therapies. Additional computational models have explored alternatively activated macrophages in tuberculosis studies [77,78,79]. These models are essential for understanding immune dynamics in granulomatous diseases but remain pathogen-specific and context-dependent.

The ongoing integration of AI and computational technologies promises to deliver increasingly sophisticated models that could revolutionize our understanding of granuloma biology. Nevertheless, these models must undergo rigorous validation using real-world samples and should complement, rather than replace, other experimental approaches.

## 3. Discussion and Future Perspectives

In vitro and in vivo models developed over time for studying granulomatous diseases have significantly advanced our understanding of key factors and biological pathways involved in cell aggregation, granuloma formation, and its persistence (Figure 1; Table 2). These include macrophages’ alternative polarization and their key role in granuloma initiation even without a T-cell influence, unraveling the importance of the mTORC1 signaling pathway and dysregulated lipid metabolism. However, T cells’ relevance in granuloma formation has also been highlighted by zebrafish KO models.

The use of primary cells from sarcoidosis patients has allowed researchers to analyze several cell populations and identify phenotypic differences compared with control subjects, also highlighting the importance of cell–cell interactions within granulomas. Additionally, the development of 2D and 3D in vitro models allows us to follow the 3Rs principle, “*replacement, reduction, refinement*”, thus providing several approaches that enable the reduction in animals’ involvement in biomedical studies.

However, research on MGCs in the granuloma core and their interactions with other cell types remains limited. Moreover, attempts to recapitulate granuloma formation in the laboratory mainly rely on specific microbial or non-organic antigens for induction, enabling the in vitro reproduction of distinct granulomatous manifestations, but possibly in an “antigen-influenced” fashion and focusing on the lung disease. For instance, carbon nanotubes and other inorganic particles can trigger cell aggregation, making them suitable for modeling granulomas formed due to environmental dust exposure. However, whether such approaches can effectively represent granuloma induction mechanisms in the absence of specific exposure and extrapulmonary tissues remains unclear. A similar concern applies to models using mycobacteria or their protein derivatives, widely accepted as granuloma inducers. Since the mechanisms by which mycobacteria induce granulomas are well-defined [24], questions arise regarding the accuracy of these models in revealing granuloma dynamics in all conditions, and mycobacteria, or eventually *P. acnes*, might not always be a suitable trigger. As shown in the paper by Crouser et al., indeed, PBMCs from 6 of 12 active pulmonary sarcoidosis patients were classified as “low-responders” to PPD exposure since they were not able to produce significant granuloma-like cell aggregates despite fulfilling the same inclusion criteria for the “high-responder group” [54]. Given the well-known genetic and exposure history heterogeneity of sarcoidosis and GLILD patients, models using a standardized trigger might, indeed, not be suitable for all uses. However, the trigger might not be the only variable to be considered, and even when using only specific cytokine combinations without antigenic stimulation to induce MGC formation (Table 2), there is a risk of obtaining a model representing only one of the multiple phases of the granulomatous inflammation. Of note, Lim et al. used for their in vitro studies sorted monocytes from chronic sarcoidosis patients with skin and/or lung disease. Apart from using GM-CSF to induce differentiation into macrophages, in their T cell-free model, they did not introduce any organic or inorganic antigen or specific cytokine, still obtaining granuloma-like structures [49]. Compared with the spheroid model by Crouser et al., the selection of primary cells from chronic instead of active sarcoidosis patients might implicate a different tendency toward spontaneous aggregation and differentiation into MGCs, which may be related to a different phase of the clinical disease and unravel different information. At the same time, the use of sorted macrophages instead of PBMCs and the absence of an antigenic trigger remove any possible bias due to a possible T-cell role, in line with the *Tsc2* KO mouse model. Meanwhile, macrophages alone in a 2D setting cannot fully recapitulate the complexity of the granulomatous microenvironment.

Apart from the trigger and the inflammatory phase, most current in vitro models also focus on the granulomas themselves and rarely consider the interplay between granuloma components and the microenvironment (Table 2). It is now clear how cellular and extracellular matrix tissue-specific factors (e.g., signals or physical properties, such as stiffness) may influence cell growth as well as the development and maintenance of inflammation and fibrosis [80,81].

Despite the considerable efforts that have been made to recreate more complex in vitro microenvironments and to incorporate organ-specific factors, these have primarily been focused on the pulmonary environment (e.g., 3D lung models with fluidic systems [32]) while other organ-specific settings, such as bones, eyes, brain, or heart, also remain underexplored in granuloma modeling (Figure 1, Table 2). This is particularly relevant when considering the impact of extrapulmonary sarcoidosis on disease prognosis and the higher lack of robust evidence in the treatment of extra-pulmonary compared with pulmonary disease [82].

A notable advancement in this field was the development of the conditional myeloid *Tsc2* KO mouse model, which spontaneously develops a systemic granulomatous disease without requiring external antigenic or T cell-mediated induction [33]. This groundbreaking model not only represents the first non-infected murine granuloma model for sarcoidosis studies but also paves the way for organ-specific research that might be compared with the tissue-specific in vitro setting [32].

Moreover, the *Tsc2* KO mouse model presents lung, liver, lymph nodes, skin, and heart granulomatous inflammation and has already been used as a platform to test the effects of mTOR inhibition as a possible treatment for sarcoidosis. Of note, a subsequent clinical trial demonstrated that the mTOR inhibitor sirolimus may be safe and effective in patients with persistent cutaneous sarcoidosis [83]

In vitro models have also been used as a platform for preliminary in vitro testing of sarcoidosis treatment before the clinical experimental phase [55,59], thus confirming the potential clinical relevance of in vivo and in vitro sarcoidosis modeling and strengthening the call for extrapulmonary disease models to test organ-specific treatment approaches, a suggested by Bueno-Beti and colleagues for cardiac sarcoidosis [72].

From this perspective, the spheroid model developed by Coruser et al. already represents a well-accepted and relatively accessible tool, while in vivo models, 3D models with organ-like scaffolds, and fluidic systems represent the most promising platforms for recapitulating the complex immunopathogenesis of granulomatous diseases [84]. Regarding the in vitro models, the chosen cell source appears to be particularly relevant, taking into account both the organ involvement and disease phase of the donors (active versus inactive and acute versus chronic), as well as treatment history. These approaches could incorporate PBMCs and sera from patients with specific organ involvement, enabling a detailed comparison of different types of granuloma, including those found in rare conditions such as GLILD [23]. Integration with omics technologies and computational/AI-based approaches has the potential to enhance our ability to interpret and validate results, paving the way for personalized disease models. Such platforms, whether in vitro or virtual, could prove invaluable in designing tailored strategies for patients with refractory disease, offering a more precise and individualized approach to therapy.

## 4. Conclusions

In conclusion, future efforts should prioritize the development of organ and disease-specific granuloma models to unravel the mechanisms behind granuloma formation and persistence. This approach could help identify potential therapeutic targets and provide platforms for testing the efficacy of targeted treatments. However, progress in granulomatous disease research remains constrained by a challenging paradox: our limited understanding of granuloma formation and persistence impedes the creation of effective models, while the lack of robust models restricts our ability to address these knowledge gaps. Breaking the cycle will require innovative strategies that integrate advanced technologies, foster interdisciplinary collaboration, and actively seek to overcome existing limitations.

## Figures and Tables

**Table 1:** Current overview of granuloma modeling.

	Setting	Induction	Specificity	Outcome	Contribution
**2D**	PBMCs/monocytes [25]	Cytokine cocktails/pathogens or parasites/chemical treatments	No	**2D MGCs**	Macrophages fusion capability and cytokine milieu evaluation
**Spheroids**	THP-1 cell line in ultra-low attachment plates [26]	*M. bovis* BCG infection	No	**3D spheroids**	Bacilli disposition in the 3D culture/anti-TB treatment screening
	Monocytes/macrophages [27]	Multi-walled carbon nanotubes	Lungs	**3D spheroids**	Nanomaterial inhalation mimicking
	PBMCs [28]	PPD-coated beads	No	**3D spheroids**	Aggregation scheme defining/gene expression investigation and pre-clinical drug testing
**ECM-Based**	PBMCs [29]	Agarose beads	No	**3D aggregates**	First phases of granuloma formation resembling
	Monocytes/macrophages and epithelial pulmonary cells [30]	*M. tuberculosis* infection and air exposure in a transwell system	Lungs	**3D granuloma-like structures**	Lung environment reproduction
	Monocytes/macrophages and ECM [31]	Bioelectrospray	No	**Cells containing** **hydrogel droplets**	Closed environment generation and physiological condition resembling
**Fluidic and Animal**	PBMCs [32]	*M. abscessus*-generated microparticles and fluidic device	Lungs	**3D granulomas in lung-on-chip device AI-controlled**	Pulmonary sarcoidosis mimicking
	Mice [33]	mTORC1 overexpression through conditional myeloid *Tsc2* deletion in a mouse model	Lungs, liver, lymph nodes, skin, heart	**Autonomously** **rising granulomas**	Autonomous granuloma formation through mTORC1 overexpression in macrophages; pre-clinical drug testing
		MWCNT instillation in a ABCG1 knockout mouse model	Lungs	**Exposure-triggered granulomas**	From chronic granulomatous inflammation to fibrosis
	Zebrafish [34]	*M. tuberculosis* or *M. marinum* infection	No	**Macrophage** **aggregation**	Granuloma-like aggregation independent from T cells

**Table 2:** In vitro MGC induction.

Cell Source	Stimuli	Outcome
Monocytes [35]	Concanavalin A-stimulated PBMC supernatant	Higher percentage of granuloma-type MGCs in comparison with controls
RAW mouse cell line [36]	Lipopolysaccharide and concanavalin A combination	MGCs with a concomitant increased production of TNF-α
Monocytes [37]	IFN- γ	MGCs with significantly increased production of H_2_O_2_ in response to PMA
Monocytes [38,39]	Concanavalin A-stimulated PBMC supernatant or IFN- γ	MGCs in higher percentage when treated with supernatant in comparison with IFN- γ alone
Monocytes [40]	IL-3 and IFN- γ combination	MGCs formed rapidly with 67 ± 6% fusion after 1 week of culture
Monocytes [25,41]	GM-CSF followed by IL-4	MGCs formed more efficiently on plasticware than untreated monocytes
Monocytes [42,43]	M-CSF followed by IL-13 or IL-4	Formation of osteoclast-like cells not properly resembling granuloma MGCs
Monocytes [44]	*M. bovis* bacillus Calmette–Guérin in addition to cytokine-containing supernatants from herpesvirus saimiri-transformed human T cells	MGCs formed with fusion rate of 27%
PBMCs [45]	Co-culture with *Nippostrongylus brasieliensis* larvae	Epithelioid cells and MGCs clustered in granulomatous-like structure
Monocytes [46]	PHA or NaIO_4_	MGCs with a morphology that was different from Langhans cells found at sites of delayed hypersensitivity reactions
Monocytes [47]	PMA in addition to IFN-γ pre-treatment	MGC induction with fusion rate increasing from 30% to 80% and enhanced by IFN-γ

## Data Availability

No new data were created or analyzed in this study. Data sharing is not applicable to this article.
